# Release of HER2 repression of trefoil factor 3 (TFF3) expression mediates trastuzumab resistance in HER2+/ER+ mammary carcinoma

**DOI:** 10.18632/oncotarget.18431

**Published:** 2017-06-09

**Authors:** Qing-Yun Chong, Ming-Liang You, Vijay Pandey, Arindam Banerjee, Yi-Jun Chen, Han-Ming Poh, Mengyi Zhang, Lan Ma, Tao Zhu, Salundi Basappa, Liang Liu, Peter E. Lobie

**Affiliations:** ^1^ Cancer Science Institute of Singapore, National University of Singapore, Singapore; ^2^ Department of Pharmacology, Yong Loo Lin School of Medicine, National University of Singapore, Singapore; ^3^ Tsinghua Berkeley Shenzhen Institute, Tsinghua University Graduate School at Shenzhen, Shenzhen, China; ^4^ Hefei National Laboratory for Physical Sciences at Microscale and School of Life Sciences, University of Science and Technology of China, Hefei, Anhui, China; ^5^ The CAS Key Laboratory of Innate Immunity and Chronic Disease, School of Life Sciences, University of Science and Technology of China, Hefei, Anhui, China; ^6^ Laboratory of Chemical Biology, Department of Chemistry, Bangalore University, Central College Campus, Bangalore, India; ^7^ Department of Oncology, Fudan University Shanghai Cancer Center, Fudan University, Shanghai, China; ^8^ Department of Radiology, Fudan University Shanghai Cancer Center, Fudan University, Shanghai, China; ^9^ National University Cancer Institute, Singapore

**Keywords:** trefoil factor 3, breast cancer, HER2, trastuzumab resistance, estrogen receptor

## Abstract

HER2+/ER+ breast cancer, a subset of the luminal B subtype, makes up approximately 10% of all breast cancers. The bidirectional crosstalk between HER2 and estrogen receptor (ER) in HER2+/ER+ breast cancer contributes to resistance towards both anti-estrogens and HER2-targeted therapies. TFF3 promotes breast cancer progression and has been implicated in anti-estrogen resistance in breast cancer. Herein, we investigated the cross-regulation between HER2 and estrogen-responsive TFF3, and the role of TFF3 in mediating trastuzumab resistance in HER2+/ER+ breast cancer. TFF3 expression was decreased by HER2 activation, and increased by inhibition of HER2 with trastuzumab in HER2+/ER+ breast cancer cells, partially in an ERα-independent manner. In contrast, the forced expression of TFF3 activated the entire HER family of receptor tyrosine kinases (HER1-4). Hence, HER2 negatively regulates its own signalling through the transcriptional repression of TFF3, while trastuzumab inhibition of HER2 results in increased TFF3 expression to compensate for the loss of HER2 signalling. In HER2+/ER+ breast cancer cells with acquired trastuzumab resistance, TFF3 expression was markedly upregulated and associated with a corresponding decrease in HER signalling. siRNA mediated depletion or small molecule inhibition of TFF3 decreased the survival and growth advantage of the trastuzumab resistant cells without re-sensitization to trastuzumab. Furthermore, TFF3 inhibition abrogated the enhanced cancer stem cell-like behaviour in trastuzumab resistant HER2+/ER+ breast cancer cells. Collectively, TFF3 may function as a potential biomarker and therapeutic target in trastuzumab resistant HER2+/ER+ breast cancer.

## INTRODUCTION

HER2/*neu*- and estrogen receptor α-positive (HER2+/ER+) breast cancer, a subset of the luminal B subtype, makes up approximately 10% of all breast cancers [1, 2]. Trastuzumab is a humanized monoclonal antibody that targets the extracellular domain of HER2, and has been approved for use in combination with chemotherapy for early and metastatic HER2+ breast cancers [3, 4]. Trastuzumab in combination with chemotherapy is currently the preferred first-line treatment option for most patients with HER2+ disease regardless of the hormone receptor status [5–7]. However, the bidirectional signalling crosstalk between HER2 and ERα in HER2+/ER+ breast cancer has been reported to contribute to resistance towards both anti-estrogen and HER2-targeted therapies, making efficacious treatment of this group of breast cancer a challenge [6, 8].

The administration of combined anti-estrogen and HER2-targeted therapies in HER2+/ER+ breast cancer has been demonstrated to improve overall response rate (ORR), clinical benefit rate (CBR) and progression-free survival (PFS) as compared to anti-estrogen alone [9–11]. However, it is argued that the conventional trastuzumab with chemotherapy is superior in terms of overall survival (OS) rate as compared to this combination therapy although there has been no direct comparison of the two treatment regimens [6, 8]. In addition, HER2+/ER+ breast cancer has recently been shown to be a heterogeneous subgroup, of which patients could benefit from trastuzumab with chemotherapy, combined anti-estrogen and HER2-targeted therapies, or even anti-estrogen alone [8, 12, 13]. The levels of ER and progesterone receptor (PgR) expression are postulated to determine patients’ response to these different therapies [8, 12, 13]. However, the current treatment strategy for HER2+/ER+ breast cancer lacks defined standards to stratify patients to their respective optimum treatment, and fails to effectively predict for therapeutic resistance [8]. These findings reinforce the need to improve the current treatment strategy for HER2+/ER+ breast cancer [8]. The identification of novel biomarkers to predict treatment efficacy, and additional therapeutic targets to overcome drug resistance in HER2+/ER+ breast cancer, will be beneficial in facilitating the development of more efficacious therapies for the heterogeneous group of HER2+/ER+ breast cancer [8].

Trefoil factor 3 (TFF3), a member of the trefoil factor family, is predominantly expressed and secreted by the goblet cells lining the mucous membrane of the gastrointestinal tract [14, 15]. TFF3 functions in epithelial restitution and maintenance of the mucosal layer [14, 16], while the aberrant deregulation of its expression and function is likely to contribute to oncogenesis [17, 18]. TFF3 is found to be upregulated in and responsible for, the development and progression of several cancers including gastric cancer [19], hepatocellular carcinoma [20–23], prostate cancer [24], cervical cancer [25], lung adenocarcinoma [26, 27] and breast cancer [28, 29]. Notably, TFF3 has been shown to promote oncogenic transformation of *immortalized* mammary epithelial cell lines [30], and to possess pro-proliferative [29], anti-apoptotic [29], anti-anoikis [29], pro-metastatic [31] and pro-angiogenic [32] properties in breast cancer. Besides being an estrogen-responsive gene, TFF3 has been shown to increase ERα transcriptional activity in breast cancer, thereby promoting estrogen-independent growth and decreasing sensitivity towards anti-estrogens [29, 33]. Moreover, it has been reported that while TFF3 is upregulated in tamoxifen [29] and aromatase inhibitor resistant breast cancers [34], the depletion or inhibition of TFF3 resulted in re-sensitization of these resistant cells to the respective anti-estrogen [29, 34].

HER2-ERα crosstalk has been postulated to be a key contributor to trastuzumab resistance, which is a major challenge in the treatment of HER2+/ER+ breast cancer [6, 8, 35]. TFF3 is estrogen-regulated and has previously been shown to activate ERα, thereby contributing to anti-estrogen resistance [29]. Therefore, we sought to determine if TFF3 possesses a cross-regulatory relationship with HER2, whether in an ERα-dependent or -independent manner. Herein, we report a novel ERα-independent mechanism of HER2-TFF3 cross-regulation. Furthermore, with the presence of this cross-regulation, we have shown that TFF3 is functionally involved in mediating acquired trastuzumab resistance in HER2+/ER+ breast cancer.

## RESULTS

### HER2 activation decreases TFF3 expression in HER2+/ER+ breast cancer cells partially in an ERα-independent manner

**Figure 1:**
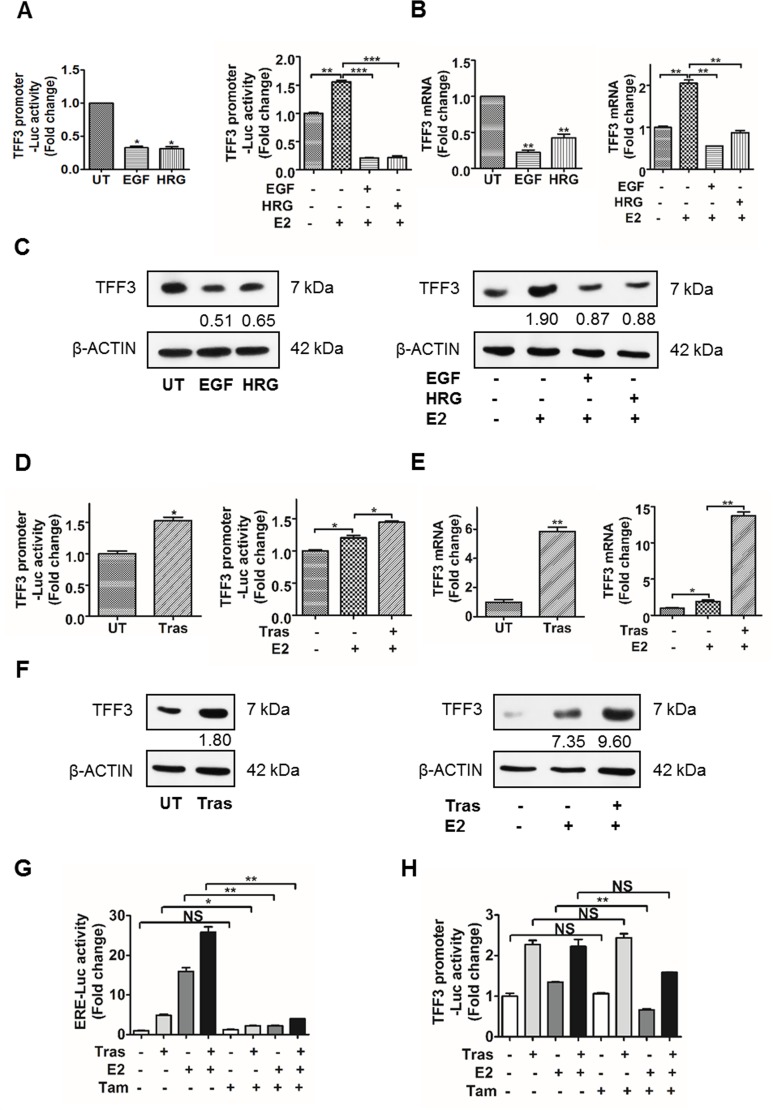
Activation of HER2 decreased TFF3 expression, while inhibition of HER2 increased TFF3 expression in BT474 cells partially in an ERα-independent manner (**A**–**C)** *Left*, BT474 cells were treated with 500 ng/ml EGF or HRG for 24 and 48 hours respectively, in phenol-red free media supplemented with 10% charcoal-stripped FBS. (A–C) *Right*, BT474 cells were treated with 200 ng/ml EGF or HRG for 48 hours in phenol-red free media supplemented with 10% charcoal-stripped FBS in the presence of 100 nM 17β-estradiol. (**D**–**F**) BT474 cells were treated with 10 µg/ml trastuzumab for 48 hours in phenol-red free media supplemented with 10% charcoal-stripped FBS ± 100 nM 17β-estradiol. (A and D) TFF3 promoter luciferase activity was measured with Renilla luciferase activity as transfection control. TFF3 (B and E) mRNA and (C and F) protein levels were determined by qPCR and western blot respectively, with β-ACTIN as input control. The densitometric analyses of protein bands were performed using ImageJ. (**G** and **H**) BT474 cells were treated with 10 µg/ml trastuzumab ± 100 nM 17β-estradiol ± 1 µM Tamoxifen for 48 hours in phenol-red free media supplemented with 10% charcoal-stripped FBS. (G) ERE and (H) TFF3 promoter luciferase activities were measured with Renilla luciferase activity as transfection control. UT: untreated; E2: 17β-estradiol; Tras: trastuzumab; Tam: tamoxifen; ERE: estrogen response element. *p < 0.05; **p < 0.01; ***p < 0.001; NS, no significance.

Given the bidirectional crosstalk between HER2 and ERα, the transcriptional regulation of estrogen-responsive TFF3 by HER2 in HER2+/ER+ breast cancer cells was investigated. Epidermal growth factor (EGF) binds EGFR, while heregulin (HRG) binds HER3 and HER4, and all three receptors dimerize with HER2 as the preferred co-receptor in HER2+ breast cancer cells, thus increasing HER2 activity [36]. In order to remove the confounding effect of estrogen-induced TFF3 expression, the experiments were performed under both estrogen-depleted and estrogen-replete conditions. We have performed time and dose-dependent analyses of the effect of EGF and HRG treatment on TFF3 expression as shown in Supplementary Figure 1. The optimum doses of EGF and HRG used in the TFF3 expression studies were 500 ng/ml under estrogen-depleted conditions, and 200 ng/ml under estrogen-replete conditions (Supplementary Figure 1A–1D, left panel). The optimum time points for EGF and HRG treatment that resulted in the greatest decrease in *TFF3* mRNA levels were 24 and 48 hours respectively under estrogen-depleted conditions (Supplementary Figure 1A and 1B, right panel). Furthermore, EGF and HRG treatment under estrogen-replete conditions were carried out for 48 hours (Supplementary Figure 1C and 1D), when the greatest E2-stimulated increase in *TFF3* mRNA levels was observed. Treatment of BT474 cells with EGF or HRG resulted in a significant decrease in TFF3 promoter luciferase activity, mRNA and protein levels under estrogen-depleted conditions (Figure 1A–1C, left panel). Exogenously administered 17β-estradiol increased TFF3 promoter luciferase activity, mRNA and protein levels, while EGF or HRG treatment markedly abrogated the 17β-estradiol-induced upregulation of TFF3 expression in BT474 cells under estrogen-replete conditions (Figure 1A–1C, right panel). Similarly, treatment with EGF or HRG led to a significant decrease in TFF3 promoter luciferase activity, mRNA and protein levels in MDA-MB-361 cells under both estrogen-depleted and estrogen-replete conditions (Supplementary Figure 2A–2C).

### HER2 inhibition by trastuzumab increases TFF3 expression in HER2+/ER+ breast cancer cells partially in an ERα-independent manner

The regulation of TFF3 expression upon HER2 inhibition by trastuzumab in HER2+/ER+ breast cancer cells was also investigated under both estrogen-depleted and -replete conditions. An optimum dose of 10 µg/ml and time point of 48 hours trastuzumab treatment were used in the TFF3 expression studies (Supplementary Figure 1E and 1F). Inhibition of HER2 by trastuzumab significantly increased TFF3 promoter luciferase activity, mRNA and protein levels in BT474 cells under estrogen-depleted conditions (Figure 1D–1F, left panel). Under estrogen-replete conditions, TFF3 promoter-luciferase activity, mRNA and protein levels were significantly increased significantly following treatment with 17β-estradiol, and were further upregulated with trastuzumab treatment in BT474 cells (Figure 1D–1F, right panel).

The transcriptional downregulation of TFF3 by HER activation and upregulation of TFF3 by trastuzumab inhibition of HER2 in BT474 cells was observed to occur partially in an estrogen-independent manner. A selective estrogen receptor modulator (SERM), tamoxifen, was used to inhibit ERα transcriptional activity to further determine the involvement of ERα in the transcriptional activation of TFF3 by trastuzumab. The effect of tamoxifen on ERα transcriptional activity at the estrogen response element (ERE) sites was also studied as a positive control. The inhibition of HER2 by trastuzumab increased ERα-mediated ERE luciferase activity in BT474 cells (Figure 1G), which is consistent with published data [37–44]. The ERα-mediated ERE-luciferase activity induced by trastuzumab, 17β-estradiol, and the combination of trastuzumab and 17β-estradiol in BT474 cells were dramatically abrogated by tamoxifen (Figure 1G). However, tamoxifen did not significantly decrease transcriptional activity of the TFF3 promoter induced by trastuzumab, and the combination of trastuzumab and 17β-estradiol in BT474 cells (Figure 1H). Hence, these results suggest that trastuzumab-stimulated TFF3 transcription in BT474 cells is partially ERα-independent.

### TFF3 mediates the activation of HER family of receptor tyrosine kinases in HER2+/ER+ breast cancer cells

**Figure 2:**
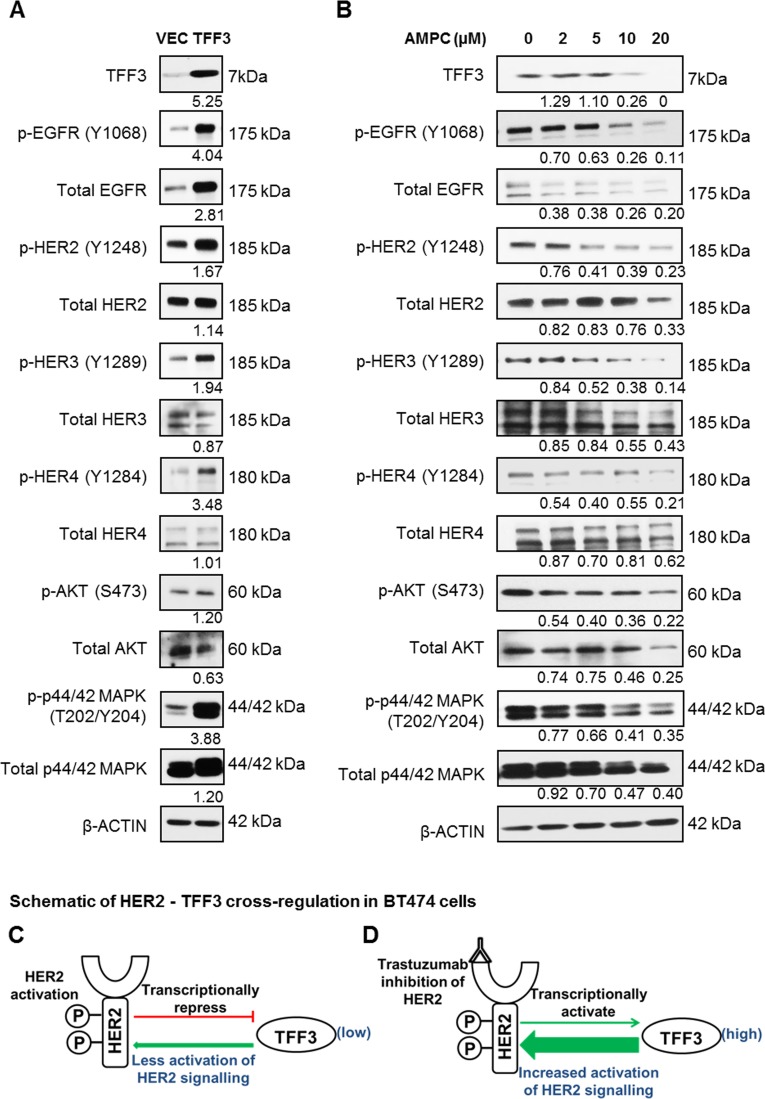
Forced expression of TFF3 activated HER signalling activity, while inhibition of TFF3 by AMPC decreased HER signalling activity in BT474 cells (**A**) BT474-VEC and -TFF3 cells were generated. (**B**) BT474 cells were treated with the indicated concentrations of TFF3 inhibitor AMPC in media supplemented with 5% FBS. Subsequently, the levels of TFF3, phosphorylated and total HER receptors and downstream signalling mediators were analysed using western blot. β-ACTIN was used as input control. The densitometric analyses of protein bands were performed using ImageJ. (**C**) Activated HER2 decreased TFF3 expression, which in turn reduced TFF3-mediated activation of HER2 signalling in a negative feedback loop. (**D**) Trastuzumab inhibition of HER2 increased TFF3 expression to compensate for the loss of HER2 signalling.

In order to determine potential TFF3 regulation of HER receptors activity, BT474 cells with stable forced expression of TFF3 were generated. The increased levels of TFF3 mRNA (Supplementary Figure 3A) and protein (Figure 2A) in BT474-TFF3 cells as compared to BT474-VEC cells were verified. The forced expression of TFF3 resulted in increased activation of the entire HER family of receptor tyrosine kinases (HER1–4) in BT474 cells, as indicated by the increased phosphorylation of the HER receptors in BT474-TFF3 cells as compared to BT474-VEC cells (Figure 2A). The total EGFR and HER2 levels were also elevated in BT474-TFF3 cells as compared to BT474-VEC cells (Figure 2A). Concordantly, the two main downstream signalling pathways of the HER receptors were activated, as characterised by an increase in phosphorylation of AKT despite decreased total AKT expression, and particularly an increase in phosphorylation of p44/42 mitogen-activated protein kinase (MAPK) associated with increased expression of p44/42 MAPK (Figure 2A).

The HER family forms a complex signalling network involving crosstalk with other receptors, thus activating a multitude of downstream effectors including STAT3 and NFκB [45]. We further investigated the effect of forced expression of TFF3 on these HER-related crosstalk pathways in BT474 cells. We observed that the forced expression of TFF3 increased activation (phosphorylation) of cMET and insulin-like growth factor 1 receptor β (IGF1Rβ) (Supplementary Figure 3B), both of which crosstalk with HER2 [46, 47]. BT474-TFF3 cells also displayed higher cSRC activation (phosphorylation of Y416) as compared to BT474-VEC cells (Supplementary Figure 3B), consistent with published literature [31]. Furthermore, STAT3 and p65 (subunit of NFκB), which are activated downstream of HER2, exhibited increased activation (phosphorylation) with forced expression of TFF3 (Supplementary Figure 3C), again in accordance with previous reports [31, 32, 48–50]. HER2 has been reported to promote cell cycle progression and enhance cell survival through the modulation of cell cycle and apoptosis regulators [51, 52]. The level of p27^Kip1^, a cyclin-dependent kinase inhibitor reported to be downregulated by HER2 [53, 54], was lower in BT474-TFF3 cells compared to BT474-VEC cells (Supplementary Figure 3D). Furthermore, the anti-apoptotic BCL-2 known to be upregulated by HER2 [55–57], increased its expression in BT474-TFF3 cells as compared to BT474-VEC cells (Supplementary Figure 3D), consistent with a previous study [29].

Conversely, the siRNA mediated depletion of TFF3 decreased the phosphorylation of HER1-4 and the downstream p44/42 MAPK (Supplementary Figure 3E). The effect of TFF3 inhibition on HER signalling was also determined using a novel small molecule TFF3 inhibitor AMPC developed in our laboratory (manuscript in preparation). AMPC treatment decreased TFF3 protein levels in a dose-dependent manner in BT474 cells, with a significant reduction of TFF3 levels at an AMPC concentration of 10 µM and higher (Figure 2B). In contrast to the effect of forced expression of TFF3, the inhibition of TFF3 by AMPC decreased HER1-4 phosphorylation in a dose-dependent manner (Figure 2B). The phosphorylation of downstream AKT and p44/42 MAPK decreased proportionately with the inhibition of TFF3 at increasing AMPC concentrations (Figure 2B). Consistent with increased total EGFR and p44/42 MAPK levels with forced expression of TFF3, they were, however, decreased with AMPC inhibition of TFF3 (Figure 2B).

TFF3 was shown herein to be transcriptionally downregulated by HER2 activation and upregulated by trastuzumab inhibition of HER2 in BT474 cells. Furthermore, we demonstrated that TFF3 mediated the activation of HER2 signalling in BT474 cells. Taken together, it is proposed that HER2 regulates its own signalling in a negative feedback loop through transcriptional repression of TFF3 in HER2+/ER+ breast cancer cells (Figure 2C). Activated HER2 decreases TFF3 levels, which in turn reduces TFF3-mediated HER2 activation. In contrast, trastuzumab inhibition of HER2 releases the repression on TFF3 expression to partially negate the loss of HER2 signalling (Figure 2D).

### TFF3 increases the 3D growth of HER2+/ER+ breast cancer cells in the presence of trastuzumab

**Figure 3:**
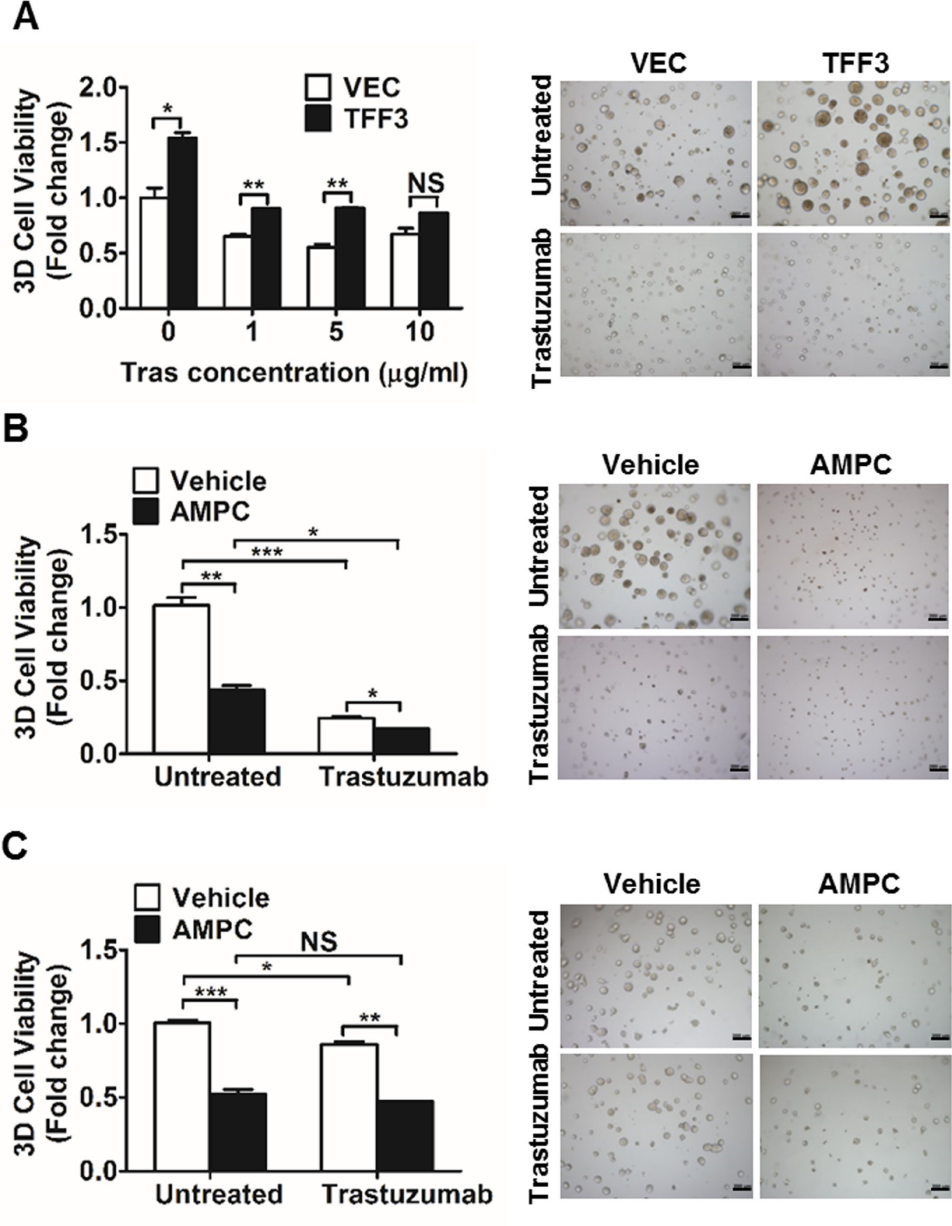
Forced expression of TFF3 increased, whereas AMPC inhibition of TFF3 decreased 3D Matrigel growth of HER2+/ER+ breast cancer cells in the presence of trastuzumab (**A**) BT474-VEC and -TFF3 cells were cultured in 5% FBS media containing 4% Matrigel and treated with the indicated concentrations of trastuzumab over a period of 8 to 10 days. Cell viability in 3D Matrigel was measured by AlamarBlue assay. (**B**) BT474 cells were cultured in 5% FBS media containing 4% Matrigel, and treated with AMPC (IC_50_ ≈ 2 µM in BT474 cells under 3D growth condition) ± 10 µg/ml trastuzumab over a period of 8 to 10 days. Cell viability in 3D Matrigel was measured by AlamarBlue assay. (**C**) MDA-MB-361 cells were cultured in 10% FBS media containing 4% Matrigel, and treated with AMPC (IC_50_ ≈ 5 µM in MDA-MB-361 cells under 3D growth condition) ± 10 µg/ml trastuzumab over a period of 8 to 10 days. Cell viability in 3D Matrigel was measured by AlamarBlue assay. Tras: trastuzumab; Vehicle: DMSO solvent for AMPC. Scale bar: 200 µm. *p < 0.05; **p < 0.01; ***p < 0.001; NS, no significance.

As we observed that TFF3 expression is HER2-regulated and activates HER signalling, we went on to determine whether TFF3 modulates trastuzumab response in HER2+/ER+ breast cancer cells. Three-dimensional (3D) cell culture is increasingly recognized as an attractive system for the study of drug response since it is more physiologically relevant, and thus is more predictive of *in vivo* functionality [58]. We hence investigated the effect of TFF3 forced expression on the trastuzumab response of BT474 cells in 3D Matrigel growth assays. Forced expression of TFF3 significantly increased the basal capacity for 3D Matrigel growth of BT474 cells (Figure 3A). In the presence of low trastuzumab concentrations of 1 and 5 µg/ml, BT474-TFF3 cells maintained significantly higher 3D Matrigel growth than BT474-VEC cells (Figure 3A). However, this growth advantage of BT474-TFF3 cells was abrogated in the presence of 10 µg/ml trastuzumab (Figure 3A).

We further examined the effect of siRNA-mediated TFF3 depletion or TFF3 inhibition on trastuzumab response in HER2+/ER+ breast cancer cells using 3D Matrigel growth assays. The decrease in TFF3 mRNA (Supplementary Figure 4A) and protein (Supplementary Figure 4B) levels upon siRNA-mediated depletion of TFF3 in BT474 cells was verified. Alternatively, BT474 and MDA-MB-361 cells were treated with the TFF3 inhibitor AMPC at approximate IC_50_ concentrations. The IC_50_ values of AMPC were determined to be 1.206 ± 0.3822 µM in BT474 cells; and 3.672 ± 0.5883 µM in MDA-MB-361 cells using 3D Matrigel growth conditions. siRNA-mediated depletion or AMPC inhibition of TFF3 significantly reduced the basal capacity for 3D Matrigel growth of BT474 cells (Supplementary Figure 4C and Figure 3B). The AMPC inhibition of TFF3 further significantly decreased the 3D Matrigel growth of trastuzumab-treated BT474 cells (Figure 3B), although the depletion of TFF3 did not further inhibit the 3D Matrigel growth of the trastuzumab-treated BT474 cells when compared to trastuzumab treatment alone (Supplementary Figure 4C). Similarly, the AMPC inhibition of TFF3 significantly decreased the 3D Matrigel growth of MDA-MB-361 cells, in addition to the inhibitory effect of trastuzumab (Figure 3C).

We have previously demonstrated that the siRNA mediated depletion or antibody inhibition of TFF3 decreased the soft agar colony formation and 3D matrigel growth of ER+ MCF7 cells treated with tamoxifen or fulvestrant [29]. As tamoxifen and trastuzumab were shown to exhibit a synergistic effect in inhibiting the growth of BT474 cells [59], we further examined the effect of AMPC inhibition of TFF3 in modulating response towards the combination of trastuzumab and tamoxifen in HER2+/ER+ breast cancer cells. The combination of trastuzumab and tamoxifen treatment markedly decreased the 3D matrigel growth of the HER2+/ER+ breast cancer cells, while the AMPC inhibition of TFF3 could further decrease the 3D matrigel growth of these treated cells (Supplementary Figure 4D and 4E).

### TFF3 expression is markedly increased in trastuzumab resistant HER2+/ER+ breast cancer cells

**Figure 4:**
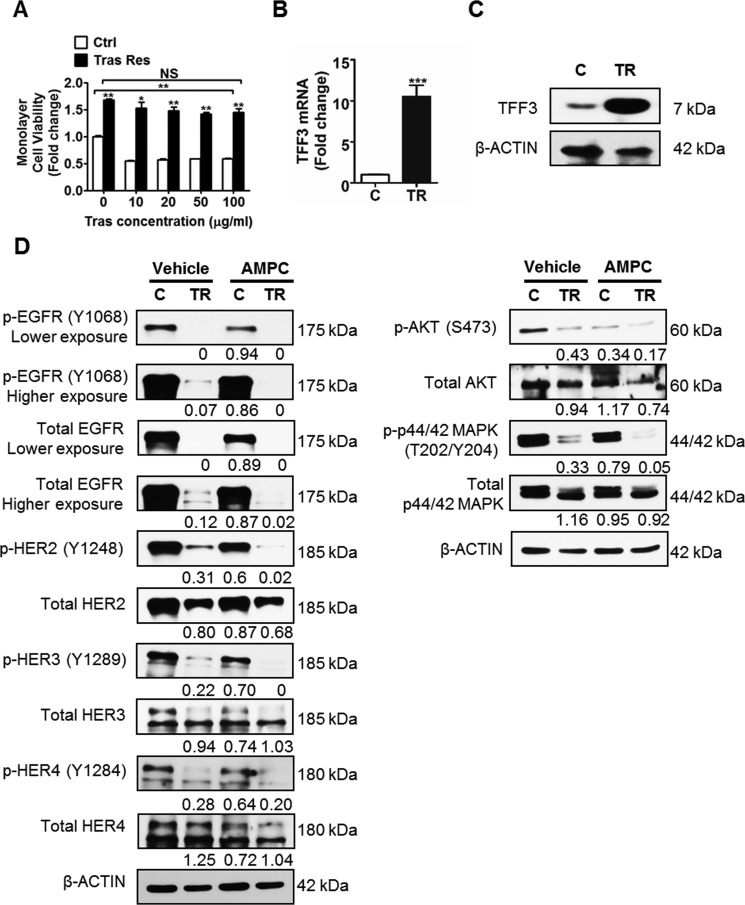
TFF3 is highly upregulated, while HER signalling is markedly decreased in trastuzumab resistant BT474 cells as compared to control cells (**A**) Control and trastuzumab resistant BT474 cells were treated with the indicated concentrations of trastuzumab in media supplemented with 5% FBS over a period of 6 days. Cell viability was measured by AlamarBlue assay. TFF3 (**B**) mRNA and (**C**) protein levels in control and trastuzumab resistant BT474 cells were determined by qPCR and western blot with β-ACTIN as input control. (**D**) Control and trastuzumab resistant BT474 cells were treated with DMSO vehicle or 20 µM AMPC for 24 hours in media supplemented with 5% FBS. The levels of phosphorylated and total HER receptors and downstream signalling mediators in the control and trastuzumab resistant cells were analysed using western blot. β-ACTIN was used as input control. The densitometric analyses of protein bands were performed using ImageJ. Ctrl or C: control cells; Tras Res or TR: trastuzumab resistant cells; Tras: trastuzumab; Vehicle: DMSO solvent for AMPC. *p < 0.05; **p < 0.01; ***p < 0.001; NS, no significance.

**Figure 5:**
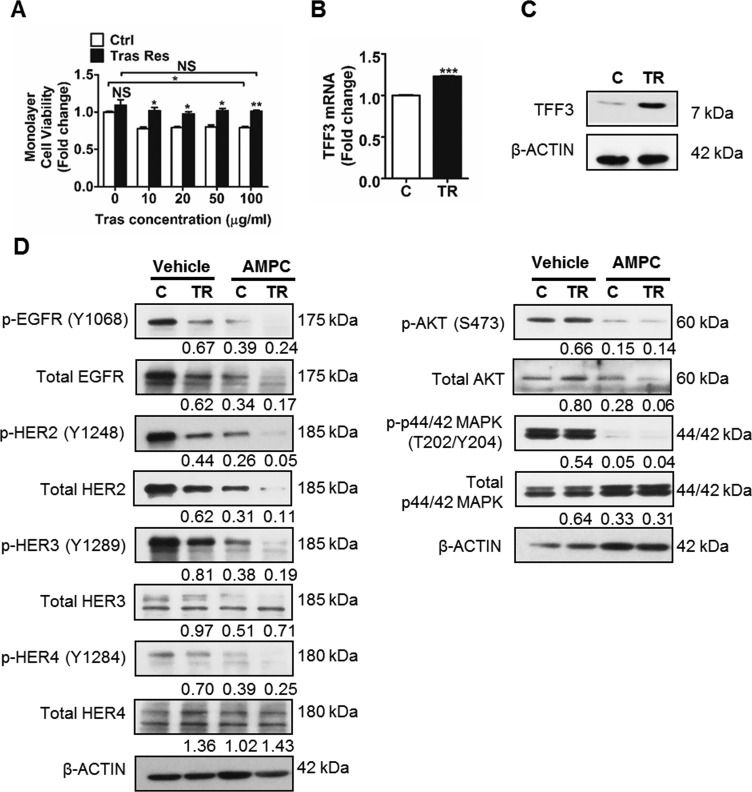
TFF3 is upregulated, while HER signalling is decreased in trastuzumab resistant MDA-MB-361 cells as compared to control cells (**A**) Control and trastuzumab resistant MDA-MB-361 cells were treated with the indicated concentrations of trastuzumab in media supplemented with 10% FBS over a period of 6 days. Cell viability was measured by AlamarBlue assay. TFF3 (**B**) mRNA and (**C**) protein levels in control and trastuzumab resistant MDA-MB-361 cells were determined by qPCR and western blot with β-ACTIN as input control. (**D**) Control and trastuzumab resistant MDA-MB-361 cells were treated with DMSO vehicle or 20 µM AMPC for 24 hours in media supplemented with 10% FBS. The levels of phosphorylated and total HER receptors and downstream signalling mediators in the control and trastuzumab resistant cells were analysed using western blot. β-ACTIN was used as input control. The densitometric analyses of protein bands were performed using ImageJ. Ctrl or C: control cells; Tras Res or TR: trastuzumab resistant cells; Tras: trastuzumab; Vehicle: DMSO solvent for AMPC. *p < 0.05; **p < 0.01; ***p < 0.001; NS, no significance.

To determine whether TFF3 mediates acquired trastuzumab resistance in HER2+/ER+ breast cancer, trastuzumab resistant BT474 and MDA-MB-361 cells were generated (Figures 4A and 5A) [60]. TFF3 mRNA and protein levels were markedly higher in trastuzumab resistant BT474 cells as compared to control BT474 cells (Figure 4B and 4C). In comparison with BT474 cells, MDA-MB-361 cells exhibited lower basal sensitivity towards trastuzumab (Figures 4A and 5A). The trastuzumab resistant MDA-MB-361 cells also exhibited upregulation of TFF3 mRNA and protein levels as compared to control MDA-MB-361 cells, albeit at a lower level (Figure 5B and 5C).

Next, we determined HER signalling status, and the interaction between TFF3 and HER signalling in trastuzumab resistant HER2+/ER+ breast cancer cells. The trastuzumab resistant BT474 cells expressed lower levels of the HER family receptors (EGFR, HER2 and HER3) as compared to control BT474 cells (Figure 4D). The levels of HER1-4 phosphorylation were also greatly reduced in the trastuzumab resistant BT474 cells, with a concordant decrease in activation of downstream AKT and p44/42 MAPK pathways (Figure 4D). The inhibition of TFF3 by AMPC eliminated the remaining low level of HER signalling activity in trastuzumab resistant BT474 cells, as indicated by a further decrease in phosphorylation of the HER family of receptors, and the downstream AKT and p44/42 MAPK (Figure 4D). The same trend of reduced HER1-4 levels and signalling activity was observed in trastuzumab resistant MDA-MB-361 cells as compared to control cells (Figure 5D). Furthermore, AMPC inhibition of TFF3 similarly decreased HER signalling in control MDA-MB-361 cells, and further reduced the low levels of HER signalling in trastuzumab resistant MDA-MB-361 cells (Figure 5D).

### Depletion of TFF3 abrogates the growth advantage of trastuzumab resistant HER2+/ER+ breast cancer cells without re-sensitization to trastuzumab

**Figure 6:**
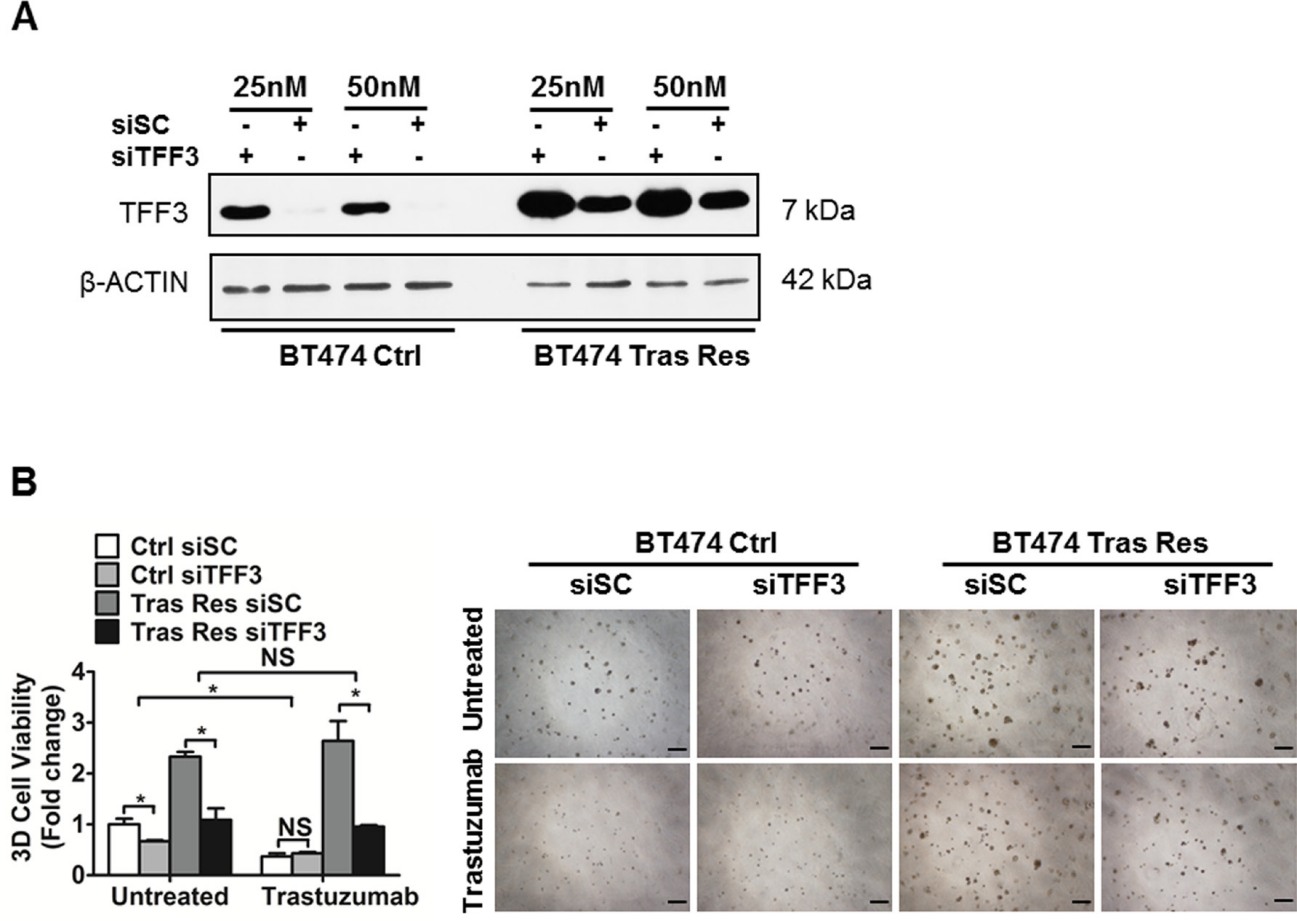
Depleted expression of TFF3 decreased 3D Matrigel growth of trastuzumab resistant BT474 cells, without re-sensitization to trastuzumab (**A**) Control and trastuzumab resistant BT474 cells were transiently transfected with the indicated concentrations of scrambled or TFF3 siRNA. TFF3 protein levels were measured with β-ACTIN as input control. (**B**) Control and trastuzumab resistant BT474 cells transfected with either scrambled or TFF3 siRNA were cultured in 5% FBS media containing 4% matrigel, and treated with 10 µg/ml trastuzumab over a period of 8 to 10 days. Cell viability in 3D Matrigel was measured by AlamarBlue assay. Ctrl: control cells; Tras Res: trastuzumab resistant cells. Scale bar, 200 µm. *p < 0.05; NS, no significance.

In order to confirm the functional roles of TFF3 in acquired trastuzumab resistance in HER2+/ER+ breast cancer cells, we depleted endogenous TFF3 in trastuzumab resistant BT474 cells with siRNA against TFF3. The level of TFF3 protein in control BT474 cells transfected with the TFF3 siRNA (siTFF3) was markedly reduced as compared to that in control BT474 cells transfected with the scrambled siRNA (siSC) (Figure 6A). However, the knockdown was less efficient in trastuzumab resistant BT474 cells, even when a higher concentration of siRNA (50 nM) was used for transfection (Figure 6A). This is attributed to the marked upregulation of TFF3 expression in trastuzumab resistant BT474 cells, and the observed resistance of these cells to transfection with trastuzumab resistant BT474 cells exhibiting a thousand times lower transfection efficiency than control BT474 cells as measured by Renilla luciferase activity.

The basal capacity for 3D Matrigel growth of trastuzumab resistant BT474 cells was markedly increased as compared to control BT474 cells (Figure 6B). The depletion of TFF3 significantly reduced the basal capacities for 3D Matrigel growth of both control and trastuzumab resistant BT474 cells (Figure 6B). In particular, the siRNA-mediated depletion of TFF3 completely abrogated the growth advantage of trastuzumab resistant BT474 cells in the 3D Matrigel growth assay without an additional inhibitory effect from trastuzumab (Figure 6B). Similarly, the depletion of TFF3 significantly decreased the 3D Matrigel growth of trastuzumab resistant MDA-MB-361 cells to the levels of the control MDA-MB-361 cells both in the absence and presence of trastuzumab (Supplementary Figure 5A).

### Inhibition of TFF3 abrogates the survival and growth advantage of trastuzumab resistant HER2+/ER+ breast cancer cells without re-sensitization to trastuzumab

**Figure 7:**
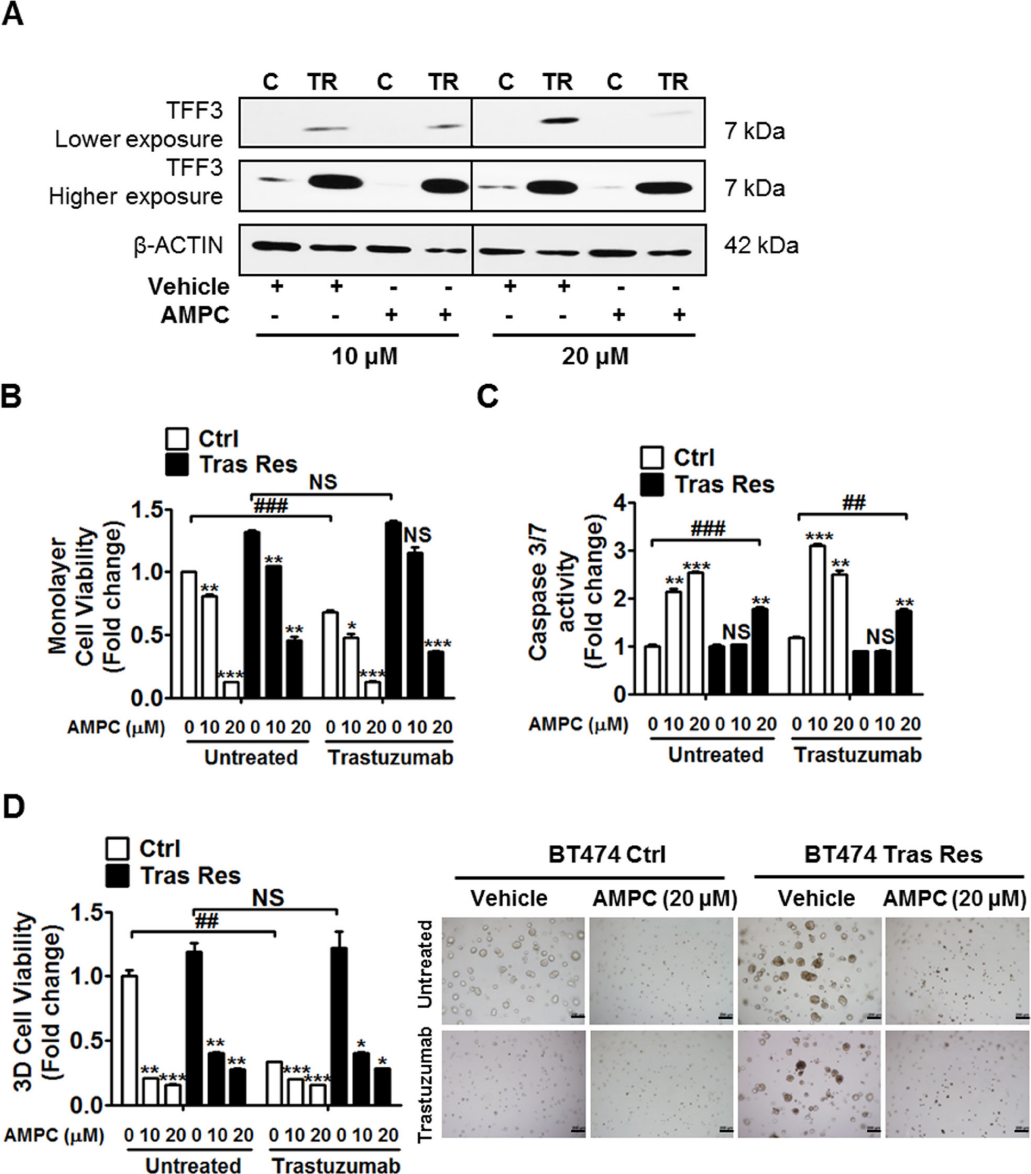
AMPC inhibition of TFF3 decreased monolayer cell viability, increased apoptosis and decreased 3D Matrigel growth of trastuzumab resistant BT474 cells, without re-sensitization to trastuzumab (**A**) Control and trastuzumab resistant BT474 cells were treated with 10 µM and 20 µM of AMPC for 24 hours in media supplemented with 5% FBS. TFF3 protein levels were measured with β-ACTIN as input control. Control and trastuzumab resistant BT474 cells were treated with the indicated concentrations of AMPC ± 10 µg/ml trastuzumab in media supplemented with 5% FBS. (**B**) Cell viability was measured by AlamarBlue assay after 6 days. (**C**) Caspase 3/7 activity was determined after 24 hours. (**D**) Control and trastuzumab resistant BT474 cells were cultured in 5% FBS media containing 4% matrigel, and treated with the indicated concentrations of AMPC ± 10 µg/ml trastuzumab, over a period of 8 to 10 days. Cell viability in 3D Matrigel was measured by AlamarBlue assay. The statistical significance in cell viability differences between the AMPC treated cells as compared to vehicle treated cells is represented by the * symbol, while that between the trastuzumab treated as compared to untreated cells is represented by the # symbol. C or Ctrl: control cells; TR or Tras Res: trastuzumab resistant cells; Vehicle: DMSO solvent for AMPC. Scale bar, 200 µm. *p < 0.05; ** or ^##^*p* < 0.01; *** or ^###^*p* < 0.001; NS, no significance.

The effect of AMPC inhibition of TFF3 on acquired trastuzumab resistance in HER2+/ER+ breast cancer cells was also investigated. AMPC treatment decreased TFF3 protein levels in control and trastuzumab resistant BT474 cells (Figure 7A). A higher concentration of AMPC (20 μM) was required to produce a significant decrease in TFF3 protein in trastuzumab resistant BT474 cells, which showed a markedly higher TFF3 expression than in control BT474 cells (Figure 7A).

Similar to depletion of TFF3, AMPC inhibition of TFF3 significantly reduced the basal monolayer cell viability of control and trastuzumab resistant BT474 cells in a dose-dependent manner (Figure 7B). AMPC at a concentration of 10 μM decreased the monolayer cell viability of control BT474 cells in addition to trastuzumab inhibition, while a higher concentration of AMPC (20 μM) was sufficient to markedly reduce the monolayer cell viability of control BT474 cells without an additional inhibitory effect from trastuzumab (Figure 7B). The trastuzumab resistant BT474 cells with elevated TFF3 expression required a higher concentration of AMPC (20 μM) for a significant reduction of monolayer cell viability in the presence of trastuzumab (Figure 7B). AMPC treatment decreased the monolayer cell viability of trastuzumab resistant BT474 cells without re-sensitizing them to trastuzumab (Figure 7B). Likewise, AMPC treatment also reduced the monolayer cell viabilities of control and trastuzumab resistant MDA-MB-361 cells, without an additional inhibitory effect from trastuzumab (Supplementary Figure 5B). The inhibition of TFF3 by AMPC reduced the monolayer cell viability of control and trastuzumab resistant BT474 cells by promotion of apoptosis. AMPC treatment significantly increased caspase 3/7 activities of control and trastuzumab resistant BT474 cells, with the trastuzumab resistant cells requiring a higher concentration of AMPC (20 μM) to stimulate a significant increase in apoptosis (Figure 7C). In addition, the inhibition of TFF3 by AMPC significantly reduced the capacity for 3D Matrigel growth of control BT474 cells and abrogated the 3D growth advantage of trastuzumab resistant BT474 cells, without an additional inhibitory effect from trastuzumab (Figure 7D). Similar results upon AMPC treatment were observed in control and trastuzumab resistant MDA-MB-361 cells (Supplementary Figure 5C).

### Inhibition of TFF3 abrogates the increased cancer stem cell (CSC)-like behaviour in trastuzumab resistant HER2+/ER+ breast cancer cells

**Figure 8:**
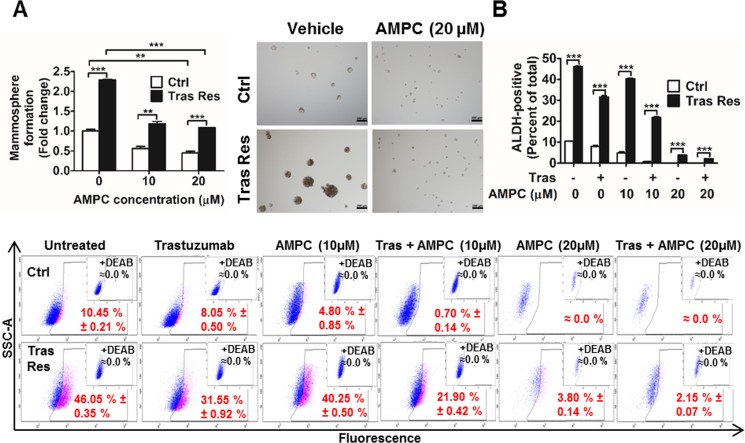
Trastuzumab resistant BT474 cells exhibited higher cancer stem cell-like behavior that was abrogated by AMPC inhibition of TFF3 (**A**) Control and trastuzumab resistant BT474 cells were seeded in ultra-low attachment plates in mammospheric growth media, and treated with the indicated concentrations of AMPC. After 10 days, mammospheric growth was measured by AlamarBlue. Representative images of mammospheres generated were shown. Scale bar, 200 µm. (**B**) Control and trastuzumab resistant BT474 cells were treated with trastuzumab or AMPC or their combination in media supplemented with 5% FBS for 4 days. The cells were then harvested and incubated with ALDEFLUOR substrate (BAAA, BODIPY^®^-aminoacetaldehyde) to define the ALDH1-positive population. A specific inhibitor of ALDH1, diethylaminobenzaldehyde (DEAB), was used to establish the baseline fluorescence. Flow cytometry plots indicate side scatter (SSC) versus fluorescence intensity. Ctrl: control cells; Tras Res: trastuzumab resistant cells; Vehicle: DMSO solvent for AMPC. **p < 0.01; ***p < 0.001.

It has previously been reported that increased CSC like behaviour plays a key role in acquired trastuzumab resistance [60, 61]. Hence, we also examined whether TFF3 modulates the CSC-like population in trastuzumab-resistant HER2+/ER+ breast cancer cells. The mammosphere-forming capacity was greatly enhanced in trastuzumab resistant BT474 cells as compared to that in control BT474 cells, as indicated by the greater number and size of viable mammospheres formed by the former under DMSO vehicle treatment (Figure 8A). However, upon inhibition of TFF3 by AMPC, the mammosphere-forming capacities of both control and trastuzumab resistant BT474 cells were greatly reduced (Figure 8A). In particular, the higher mammosphere-forming capacity of the trastuzumab resistant cells was abrogated.

ALDEFLUOR^™^ assay is often used to detect cells that express the cancer stem cell marker aldehyde dehydrogenase-1 (ALDH1). This assay was also performed to study the effect of AMPC on cancer stem cell-like behaviour in control and trastuzumab resistant BT474 cells. The trastuzumab resistant BT474 cells exhibited a markedly higher ALDH1-positive population than control BT474 cells (Figure 8B). Trastuzumab or AMPC treatment alone significantly decreased the ALDH1-positive population in both control and trastuzumab resistant BT474 cells (Figure 8B). Furthermore, the combination of trastuzumab and AMPC acted synergistically to effectively eliminate the ALDH1-positive population in control BT474 cells and abrogated the elevated ALDH1-positive population in trastuzumab resistant BT474 cells (Figure 8B). Therefore, besides inhibiting survival and growth, AMPC treatment also depleted the cancer stem cell-like population in trastuzumab resistant BT474 cells. AMPC is hence useful in targeting both the bulk population of non-stem cancer cells and the increased cancer stem cell population in HER2+/ER+ breast cancer cells with acquired trastuzumab resistance.

## DISCUSSION

HER2+/ER+ breast cancer is characterized by HER2-ERα signalling crosstalk, and is prone to resistance towards both anti-estrogen and HER2-targeted therapies [6, 8]. As TFF3 is an estrogen-responsive oncogene [33, 62], our study builds on the existing HER2-ERα crosstalk and established the cross-regulation between HER2 and TFF3 in an ERα-independent manner. Previously, the cross-regulation between ERα and TFF3 has implicated TFF3 in anti-estrogen resistance [29]. In this study, we showed that the cross-regulation between HER2 and TFF3 identifies TFF3 as a mediator of trastuzumab resistance in HER2+/ER+ breast cancer.

Herein, we demonstrated that TFF3 is transcriptionally downregulated by HER2 activation and upregulated by trastuzumab inhibition of HER2 in HER2+/ER+ breast cancer cells. Previously, we observed TFF3 expression to be associated with low HER2 levels in a mammary carcinoma patient cohort, although the difference was not statistically significant [31]. Furthermore, previous studies have reported a negative correlation of TFF3 level and HER2 status, in which TFF3 was found to be more frequently expressed in gastric tumours without HER2 amplification as compared to those with HER2 amplification [63, 64]. Our current findings are consistent with these observations in that HER2 transcriptionally represses TFF3, while trastuzumab inhibition of HER2 releases the transcriptional repression on TFF3 in HER2+/ER+ breast cancer cells. In contrast to our findings of trastuzumab-stimulated upregulation of TFF3 in BT474 cells, Yue *et al.* observed that the silencing of HER2 induced TFF3 downregulation in HER2+/ER− SKBR3 cells [65]. This discrepancy may be attributed to two reasons. Firstly, the mechanisms of HER2 transcriptional regulation of TFF3 in different breast cancer subtypes are probably varied. We found that TFF3 is negatively regulated by HER2 and is employed by HER2 to maintain the appropriate level of HER signalling in HER2+/ER+ breast cancer cells. However, in HER2+/ER− SKBR3 cells that express relatively low levels of TFF3, it is possible that HER2 may not depend on transcriptional regulation of TFF3 to regulate its own signalling. Secondly, trastuzumab inhibition of HER2 possibly exerts a distinct effect as compared to endogenous knockdown of HER2 since trastuzumab was previously confirmed to enhance HER2 phosphorylation at specific phosphotyrosine sites in trastuzumab-sensitive breast cancer cells [66–68]. It will be necessary to determine whether decreased HER2 levels and signalling can transcriptionally activate TFF3 in HER2+/ER+ breast cancer cells in general or whether this is a trastuzumab-dependent effect. Specifically, the effect of siRNA-mediated knockdown or lapatinib inhibition of HER2 on TFF3 expression in BT474 and MDA-MB-361 cells can be examined. NFκB is activated by HER2 and is known as a negative transcriptional regulator of TFF3, making it a potential mediator of HER2-regulated TFF3 repression [69–71]. It has been demonstrated that the NFκB binding site at position −97 of the TFF3 promoter is essential for the transcriptional downregulation of TFF3 by NFκB [69].

TFF3 has previously been reported to induce tyrosine phosphorylation and activation of EGFR and HER2 in a colonic epithelial cell line (HT-29), gastric cancer cell line (AGS) and oral keratinocytes [72, 73]. Furthermore, TFF3 has been shown to activate cSRC in MCF7 breast cancer cells, which could in turn mediate EGFR phosphorylation [31, 74]. Although TFF3-mediated activation of HER3 and HER4 have not been reported, these receptors can dimerize with EGFR and HER2 and be activated [36]. As such, our study demonstrated that the forced expression of TFF3 increased, while the depletion or inhibition of TFF3 decreased, phosphorylation of the HER family of receptors in HER2+/ER+ breast cancer cells. Despite studies showing TFF3-stimulated EGFR activation, all attempts have failed to demonstrate direct binding and co-localisation of TFF3 and EGFR [75, 76]. A possible mechanism through which TFF3 activates HER2 is transactivation by crosstalk pathways including cSRC since TFF3 was previously shown to activate cSRC, a known crosstalk partner of HER2 [31, 77, 78]. There is also substantial evidence that TFF3 activates downstream AKT and p44/42 MAPK pathways [72, 73, 79–82]. Consistent with published literature, TFF3 has been demonstrated to increase phosphorylation of AKT and p44/42 MAPK in HER2+/ER+ breast cancer cells in this study. Besides the activation of HER family of receptor tyrosine kinases, it was demonstrated that TFF3 activates HER family crosstalk partners including cMET and IGF1R. cMET is a receptor tyrosine kinase, which is implicated in enhanced cell proliferation, angiogenesis, invasion and metastasis of tumour cells [83]. IGF1R is another transmembrane tyrosine kinase that is critical for cell proliferation, growth, survival and motility [84]. The capacity of TFF3 to activate cMET and IGF1R as newly discovered mechanisms of TFF3 signalling further unveils the importance of TFF3 as a therapeutic target. In addition, our observation of TFF3-mediated activation of cSRC, STAT3 and p65 (subunit of NFκB) in HER2+/ER+ breast cancer cells is consistent with published literature [31, 32, 48–50].

As a result of the cross-regulation between TFF3 and HER2, we observed that TFF3 partially decreased trastuzumab response in HER2+/ER+ breast cancer cells. Both TFF3 and trastuzumab regulate HER2 activity, albeit in opposing directions. Forced expression of TFF3 in BT474 cells increased HER2 signalling that is in turn inhibited by trastuzumab. This accounts for the observation that TFF3 forced expression only decreased the inhibitory effect of trastuzumab at low concentrations (≤ 5 µg/ml). The TFF3-mediated HER2 signalling was insufficiently inhibited at these low trastuzumab concentrations, resulting in higher 3D growth of BT474-TFF3 cells as compared to BT474-VEC cells. In contrast, a higher concentration of trastuzumab (10 µg/ml) could inhibit TFF3-mediated HER2 signalling and decrease the 3D growth of BT474-TFF3 cells to the same level as that of BT474-VEC cells. Conversely, the inhibition of TFF3, along with trastuzumab, decreased HER2 activity to achieve a greater HER2 inhibitory effect. Nevertheless, TFF3 depletion or inhibition only moderately reduced the survival and growth of the HER2+/ER+ cells in the presence of trastuzumab as TFF3 activates HER pathway, the same pathway that trastuzumab targets.

In our model of acquired trastuzumab resistance in HER2+/ER+ breast cancer, the expression of HER2 and other members of the HER family was markedly downregulated, while TFF3 was highly upregulated. The decrease or loss of HER2 following trastuzumab treatment has previously been reported in a subset of patients in the clinical settings, where HER2 status was evaluated in paired pre-treatment and post-treatment breast tumours using either immunohistochemistry (IHC) or fluorescence *in situ* hybridisation (FISH) [85–89]. Moreover, loss of HER2 amplification was observed to be associated with a poorer relapse free survival (RFS) of the trastuzumab-treated breast cancer patients in a median follow-up study [88]. Our results are consistent a previous study, whereby BT474 cells with acquired resistance to trastuzumab lost both HER2 amplification and over-expression [88]. Therefore, our findings, along with available clinical evidence, suggest that it may be useful to re-assess HER2 status in recurrent disease and that not all patients will necessarily benefit from the one-year course of adjuvant trastuzumab [88]. Nevertheless, as described above, not all HER2+ breast cancer that progresses on trastuzumab lose HER2 dependence [85, 87–89]. Furthermore, other studies have reported the maintenance or upregulation of HER signalling in acquired trastuzumab resistance [90, 91]. Herein, we observed that the trastuzumab resistant HER2+/ER+ breast cancer cells displayed a shift in oncogene addiction away from HER2 to TFF3 with highly upregulated TFF3 expression and downregulated HER signalling. In a neoadjuvant paclitaxel, trastuzumab, carboplatin combination (PTC) trial (GSE41656), TFF3 gene expression was observed to be significantly higher in non-complete responders (non-CR) of the combination of trastuzumab and chemotherapy treatment as compared to complete responders (CR) among both HER2+/ER+ and HER2+/ER- breast cancer patients (Supplementary Figure 6). To date, no clinical study has evaluated the expression of TFF3 in HER2+/ER+ breast cancers which have progressed on trastuzumab. Notably, it can be determined if there exists a clinical correlation between the loss of HER2 and increased TFF3 expression in trastuzumab resistant HER2+/ER+ breast cancer. The loss of HER2 and upregulation of TFF3 may serve as potential biomarkers to select for trastuzumab-refractory HER2+/ER+ breast cancer patients who may benefit from therapeutic inhibition of TFF3.

Collectively, our results suggest that TFF3 functionally mediates trastuzumab resistance in HER2+/ER+ breast cancer cells. The shift in oncogene addiction from HER2 to TFF3 provides justification for the use of TFF3 inhibition to overcome trastuzumab resistance. At a higher concentration, AMPC was able to abrogate the survival and growth advantage of trastuzumab resistant HER2+/ER+ breast cancer cells to a level similar to that of control cells. In addition, we observed that the high level of TFF3 in trastuzumab resistant HER2+/ER+ breast cancer cells maintains the activity of the remaining HER receptors, concordant with our finding that TFF3 activates HER signalling. Hence, AMPC inhibition of TFF3 has the added advantage of abrogating the remaining low level of HER signalling in the trastuzumab resistant HER2+/ER+ breast cancer cells such that eradication of these resistant cells did not require re-sensitization to trastuzumab. Trastuzumab tends to show higher efficacy *in vivo*, the effect being attributed to the antibody-dependent cellular cytotoxicity (ADCC) response [92]. It has been previously reported that trastuzumab resistant breast cancer cells *in vitro*, which retained HER2 expression, remained sensitive to trastuzumab *in vivo* through trastuzumab-mediated ADCC [93]. However, the marked downregulation of HER2 in our trastuzumab resistant HER2+/ER+ breast cancer cells implies that the resistant cells would probably remain relatively insensitive to trastuzumab *in vivo* due to the failure of ADCC. Nevertheless, trastuzumab resistant HER2+/ER+ breast cancer cells, which have lost HER2 expression but exhibited upregulated TFF3, could potentially be targeted by TFF3 inhibition *in vivo*. This further reinforces the importance of therapeutic TFF3 inhibition, which can eradicate the trastuzumab resistant HER2+/ER+ breast cancer cells alone without the need for trastuzumab re-sensitization.

Lastly, increasing evidence has shown that trastuzumab resistance is associated with increased breast CSC population [60, 61]. Consistently, our study also demonstrated a marked increase in CSC-like behaviour, as characterized by an increase in mammosphere-forming capacity and increased ALDH1-positive population in trastuzumab resistant BT474 cells. We have previously shown that TFF3 is responsible for the increased CSC-like population in HCC cells with acquired chemoresistance [23]. Herein, we have provided further evidence of the role of TFF3 in promoting CSC-like behaviour in trastuzumab resistant HER2+/ER+ breast cancer. Furthermore, we observed decreased cancer stem cell-like population in HER2+/ER+ breast cancer cells with trastuzumab treatment, which is consistent with published literatures [94]. Although trastuzumab treatment did not produce additional survival and growth inhibitory effects on AMPC-treated trastuzumab resistant cells, it was observed that trastuzumab and AMPC act synergistically to decrease the CSC-like population in the trastuzumab resistant HER2+/ER+ breast cancer cells. It remains to be determined whether unlike the bulk population of differentiated cancer cells that lost HER2, the CSC-like population retains high HER2 expression, rendering them to be more responsive to trastuzumab [95].

In summary, we revealed a novel mechanism of bidirectional cross-regulation between HER2 and TFF3, which is at least partially ERα-independent. Furthermore, we showed that TFF3 mediates trastuzumab resistance in HER2+/ER+ breast cancer with decreased HER2 expression and signalling. A novel small molecule TFF3 inhibitor eradicated the trastuzumab resistant HER2+/ER+ breast cancer cells, without the need for trastuzumab re-sensitization. Hence, we propose TFF3 as a potential biomarker and therapeutic target in trastuzumab resistant HER2+/ER+ breast cancer.

## MATERIALS AND METHODS

### Cell culture, transfection and trastuzumab resistant cells generation

The human HER2+/ER+ breast cancer cell lines, BT474 and MDA-MB-361, were obtained from the American Type Culture Collection (ATCC, Rockville, MD, USA) and were cultured in conditions as recommended. BT474 cells with forced expression of TFF3 were generated by transfection with empty pIRESneo3 vector or pIRESneo3-TFF3 plasmid as previously described [29]. After 4 weeks of selection in medium containing 800 μg/ml G418, the stably transfected cells were designated as BT474–VEC and BT474–TFF3 cells respectively. The siRNA oligos, namely Silencer^®^ Select Negative Control No. 1 siRNA and Silencer^®^ Select TFF3 siRNA (s277470 and s14041), were obtained from Thermo Fisher Scientific (Waltham, MA). BT474 cells with transient depletion of TFF3 were generated by transfection of the cells with TFF3 siRNA, or scrambled siRNA as a negative control, using Lipofectamine RNAiMAX (Thermo Fisher Scientific, Waltham, MA) according to the recommended protocol. The transiently transfected cells were designated as BT474-siSC and BT474-siTFF3 cells respectively. The trastuzumab resistant BT474 and MDA-MB-361 cells were generated as previously described [60]. The parental cell lines were constantly treated with 10 μg/ml trastuzumab for a period of six months until significant trastuzumab resistance was observed in the cell viability assay. Control cells were maintained alongside with normal media.

### Reagents

Trastuzumab (Herceptin) was kindly provided by Dr Wang Lingzhi from the National University Hospital (NUH), Singapore. Epidermal growth factor (EGF), 17β-estradiol, tamoxifen and bovine insulin were purchased from Sigma Aldrich (St Louis, MO). Heregulin was purchased from Calbiochem (San Diego, CA). B27 supplement and basic FGF (bFGF) were purchased from Thermo Fisher Scientific (Waltham, MA).

### Luciferase reporter assay

The estrogen response element (ERE) luciferase reporter plasmid (pGL2-ERE) used was previously described [29]. The TFF3 promoter luciferase reporter plasmid used consists of the human TFF3 promoter (−702 to +63) cloned into pGL3-basic vector. To generate the estrogen-depleted experimental condition, the cells were grown in phenol red-free media supplemented with charcoal-stripped FBS for 4 days before experiments. Luciferase assays were performed using the Dual-Luciferase^®^ Reporter Assay System (Promega, Madison, WI) as previously described [31]. Briefly, the pGL3-ERE or pGL3-TFF3 promoter luciferase reporter plasmid was co-transfected with pRL Renilla luciferase control reporter vector into the cells. 24 hours following transfection, the cells were treated with the indicated concentrations of EGF, HRG or trastuzumab in the absence or presence of 100 nM 17β-estradiol or 1 μM tamoxifen in phenol red-free media containing full charcoal stripped-serum for the respective time points before luciferase activities were assayed.

### Quantitative PCR (qPCR) analysis

qPCR was performed as described earlier [29]. The primers used for qPCR were as follows: *TFF3* forward 5′-CTTGCTGTCCTCCAGCTCT-3′, *TFF3* reverse 5′- CCGGTTGTTGCACTCCTT-3′, β*-Actin* forward 5′- GCACTCTTCCAGCCTTCCTT-3′ and β*-Actin* reverse 5′- GCACTCTTCCAGCCTTCCTT-3′.

### Western blot analysis/immunoblotting

Cells were lysed in RIPA buffer and proteins in the cell lysate were resolved using sodium dodecyl sulfate (SDS) polyacrylamide gel electrophoresis (PAGE). Western blot analysis was performed as previously described [29]. The primary antibodies used are as follows: phospho-AKT1 S473 (Abcam ab66138) [96], pan-AKT (Abcam ab8805) [97], β-Actin (Santa Cruz sc-47778), BCL-2 (Santa Cruz sc-509), phospho-cMET Y1234/1235 (Cell Signaling 3077S), cMET (Cell Signaling 8198S), phospho-cSRC Y416 (Santa Cruz sc-101802), cSRC (Santa Cruz sc-8056), phospho-EGFR Y1068 (Cell Signaling 3777S) [98], EGFR (Abcam ab2430) [99], phospho-p44/42 MAPK T202/Y204 (Cell Signaling 4370S) [100], p44/42 MAPK (Cell Signaling 4695S) [101], phospho-HER2/*neu* Y1248 (Santa Cruz sc-12352-R) [60], HER2/*neu* (Abcam ab2428) [102], phospho-HER3 Y1289 (Cell Signaling 4791S) [98], HER3 (Santa Cruz sc-415) [103], phospho-HER4 Y1284 (Cell Signaling 4757S) [104], HER4 (Santa Cruz sc-8050) [105], phospho-IGF1Rβ Y1135/1136 (Cell Signaling 3024S), IGF1Rβ (Cell Signaling 9750S), p27^kip1^ (Cell Signaling 3686S), phospho-p65 S536 (Cell Signaling 3033S), p65 (Cell Signaling 4764S), phospho-STAT3 Y705 (Cell Signaling 9131S), STAT3 (Cell Signaling 9139S), and TFF3 (Abcam ab108599) [25]. The secondary anti-rabbit, anti-mouse and anti-goat horseradish peroxidase (HRP)-conjugated antibodies were obtained from Cell Signaling Technology.

### Cell function assays

The monolayer cell viability, apoptosis and three-dimensional (3D) Matrigel growth assays were performed as previously described [29]. The cell function assays with trastuzumab treatment were carried out in reduced serum media as previously described [106, 107]. In the monolayer cell viability assay, cells were seeded at 5000 cells per well and treated with respective drugs in reduced serum media for 6 days. Cell viability was measured using AlamarBlue assay (Thermo Fisher Scientific, Waltham, MA). Apoptotic cell death was measured 24 hours after drug treatment using the Caspase-Glo^®^ 3/7 Assay kit (Promega Madison, WI) according to the manufacturer’s protocol. In the 3D Matrigel growth assay, cells were seeded at 1000 cells per well in media supplemented with 5% FBS and 4% Matrigel onto the solidified Matrigel base. The seeded cells were allowed to grow into 3D colonies in the Matrigel for 4 days before respective treatments were added. The number and sizes of the colonies were visualized under the microscope, and the viability of the colonies was determined using the AlamarBlue assay after 8–10 days.

### Cancer stem cell (CSC) assay

The mammosphere formation and ALDEFLUOR assays were performed as described previously [60]. In the mammosphere formation assay, single cells were grown in CSC growth media, which consists of serum-free DMEM/F12 1:1 media supplemented with penicillin-streptomycin, 10 ng/ml recombinant human basic FGF, 20 ng/ml recombinant human EGF, 2% B27 and 5 μg/ml bovine insulin, at a density of 500 cells per well in ultra-low attachment 96-well plate. Mammosphere formation was observed after 7–10 days and mammospheres of greater than 60 µm were counted under the microscope. The viability of the mammospheres was determined using the AlamarBlue assay.

The ALDEFLUOR assay was performed using the ALDEFLUOR assay kit (STEMCELL Technologies, USA) according to the manufacturer’s protocol. Cells were treated with respective drugs for 4 days in reduced serum media. The resulting harvested cells were incubated with ALDEFLUOR substrate (BAAA, BODIPY^®^-aminoacetaldehyde) to define the ALDH1 positive population, while a specific inhibitor of ALDH1, diethylaminobenzaldehyde (DEAB), was used to establish the baseline fluorescence.

### Statistical analysis

GraphPad Prism 5 (GraphPad Software, Inc., CA, USA) was used to generate graphical presentations and for statistical analysis. All experiments were performed at least three times and numerical data are expressed as mean ± standard deviation (SD) from a representative experiment performed in triplicate. Statistical significance was assessed using an unpaired two-tailed Student’s *t* test with *p* < 0.05 being considered as statistically significant.

## SUPPLEMENTARY MATERIALS FIGURES


