# Supplementary material for: Release of HER2 repression of trefoil factor 3 (TFF3) expression mediates trastuzumab resistance in HER2+/ER+ mammary carcinoma
Source: Oncotarget. 2017 Jun 9;8(43):74188–208. doi: 10.18632/oncotarget.18431 (PMC5650333; doi:10.18632/oncotarget.18431)
Supplement: Supplementary file 1 [file oncotarget-08-74188-s001.pdf]

Release of HER2 repression of trefoil factor 3 (TFF3) expression mediates trastuzumab resistance in HER2+/ER+ mammary carcinoma

Supplementary Materials

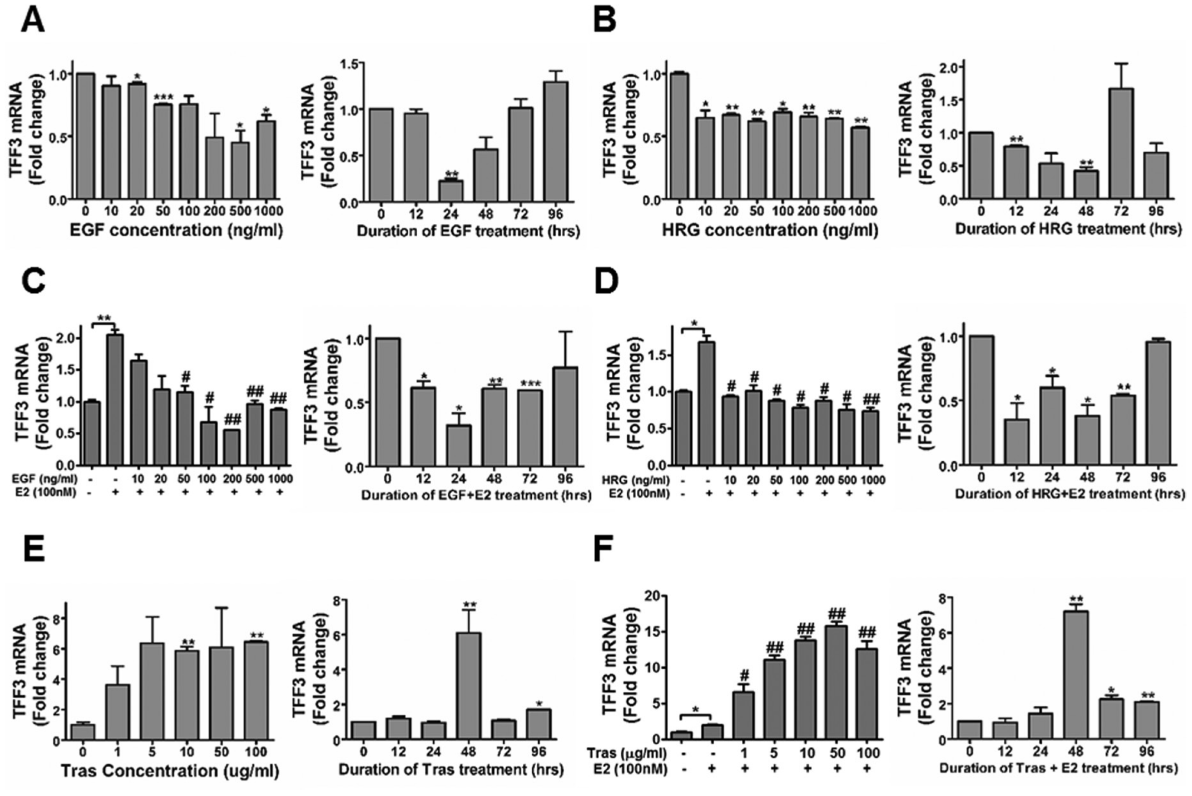

Supplementary Figure 1: Activation of HER2 decreased TFF3 expression, while inhibition of HER2 increased TFF3 expression in BT474 cells, in a dose- and time-dependent manner. (**A** and **B**) *Left*, BT474 cells were treated with the indicated concentrations of EGF or HRG for 24 and 48 hours respectively in phenol-red free medium supplemented with 10% charcoal-stripped FBS. *Right*, BT474 cells were treated with 500 ng/ml of EGF or HRG for the indicated time-points in phenol-red free medium supplemented with 10% charcoal-stripped FBS. (**C** and **D**) *Left*, BT474 cells were treated with the indicated concentrations of EGF or HRG for 48 hours in phenol-red free medium supplemented with 10% charcoal-stripped FBS and exogenous 100 nM 17β-estradiol. *Right*, BT474 cells were treated with 200 ng/ml of EGF or HRG for the indicated time-points in phenol-red free medium supplemented with 10% charcoal-stripped FBS and exogenous 100 nM 17β-estradiol. (**E** and **F**) *Left* BT474 cells were treated with the indicated concentrations of trastuzumab for 48 hours in phenol-red free medium supplemented with 10% charcoal-stripped FBS ± 100 nM 17β-estradiol. *Right*, BT474 cells were treated with 10 µg/ml of trastuzumab for the indicated time-points in phenol-red free medium supplemented with 10% charcoal-stripped FBS ± 100 nM 17β-estradiol. *TFF3* mRNA level was determined by qPCR, with *β-ACTIN* as input control. E2: 17β-estradiol; Tras: trastuzumab. In (C), (D) and (F), the statistical significance in differences in *TFF3* mRNA levels between the E2 treated cells (2nd bar) as compared to untreated cells (1st bar) is represented by the * symbol. The statistical significance of differences in *TFF3* mRNA levels between cells treated with EGF or HRG in the presence of exogenous E2 as compared to cells treated with only E2 (2nd bar) is represented by the # symbol. * or $^{\#}p < 0.05$; ** or $^{\#\#}p < 0.01$; ***$p < 0.001$.

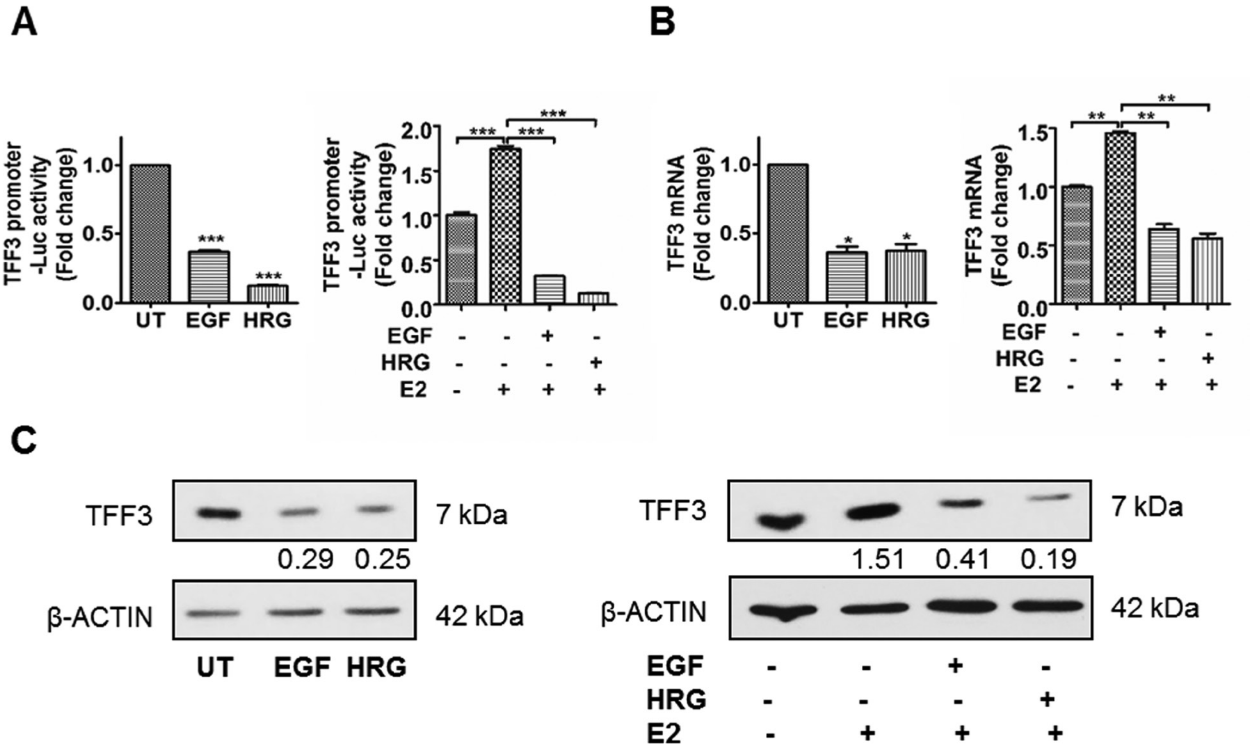

Supplementary Figure 2: Activation of HER2 decreased TFF3 expression in MDA-MB-361 cells partially in an estrogen-independent manner. (**A–C**) *Left*, MDA-MB-361 cells were treated with 500 ng/ml EGF or HRG for 24 and 48 hours respectively, in phenol-red free media supplemented with 20% charcoal-stripped FBS. (A–C) *Right*, MDA-MB-361 cells were treated with 200 ng/ml EGF or HRG for 48 hours in phenol-red free media supplemented with 20% charcoal-stripped FBS in the presence of 100 nM 17β estradiol. (A) TFF3 promoter luciferase activity was measured with Renilla luciferase activity as transfection control. TFF3 (B) mRNA and (C) protein levels were determined by qPCR and western blot respectively, with β-ACTIN as input control. The densitiometric analyses of protein bands were performed using ImageJ. *$p < 0.05$; **$p < 0.01$; ***$p < 0.001$.

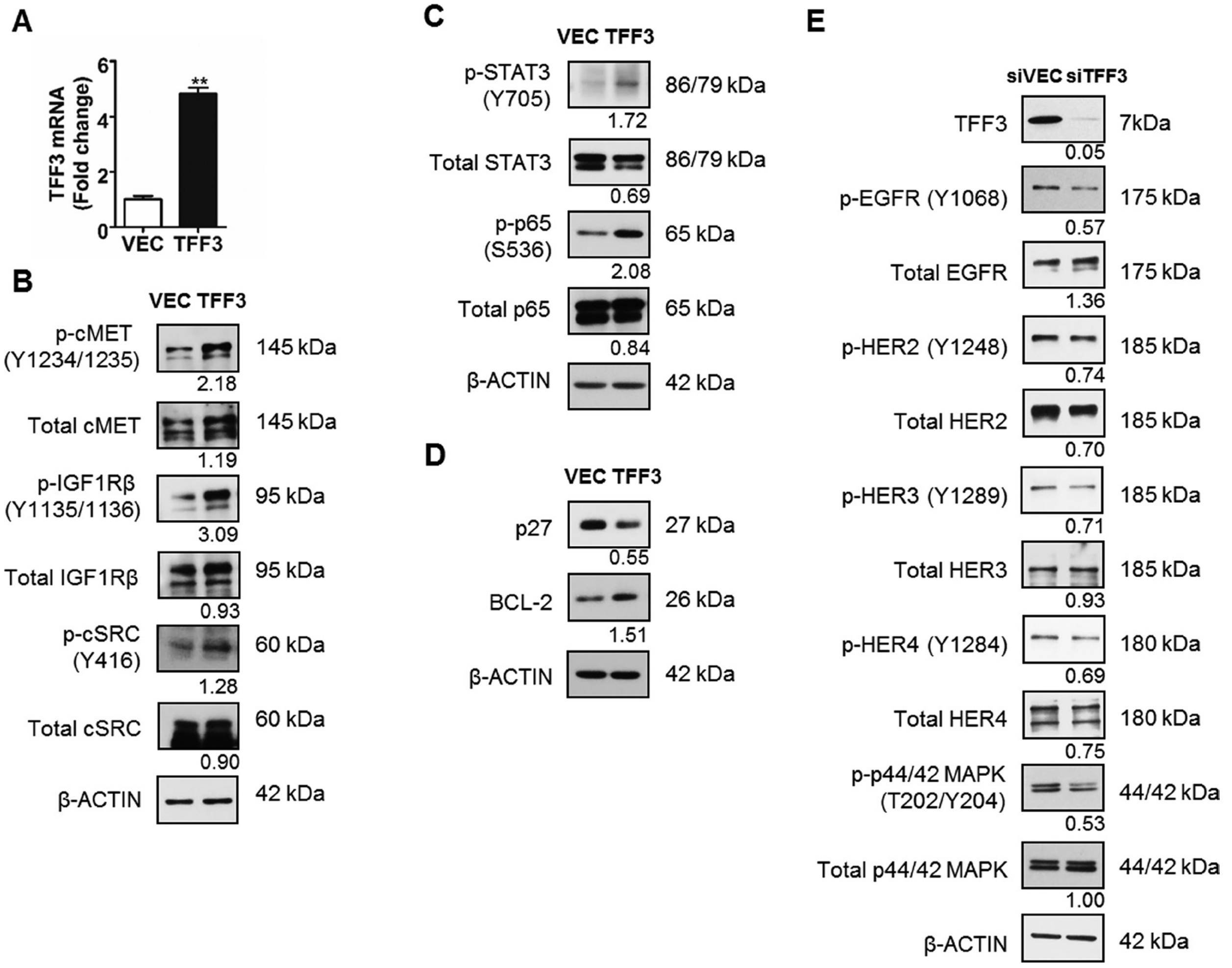

Supplementary Figure 3: Forced expression of TFF3 activated HER crosstalk pathways and HER-activated downstream mediators while depletion of TFF3 decreased HER signalling activity in BT474 cells. (**A**) *TFF3* mRNA levels in BT474-VEC and -TFF3 cells were validated by qPCR with *β-ACTIN* as input control. The levels of phosphorylated and total (**B**) HER receptors crosstalk partners and (**C**) downstream transcription factors, and the levels of (**D**) cell cycle and apoptosis regulators in BT474-VEC and -TFF3 cells were analysed by western blot. β-ACTIN was used as input control. (**E**) BT474-siSC and -siTFF3 cells were generated. The levels of TFF3, phosphorylated and total HER receptors and downstream signalling mediators were analysed using western blot. β-ACTIN was used as input control. The densitiometric analyses of protein bands were performed using ImageJ. $^{**}p < 0.01$.

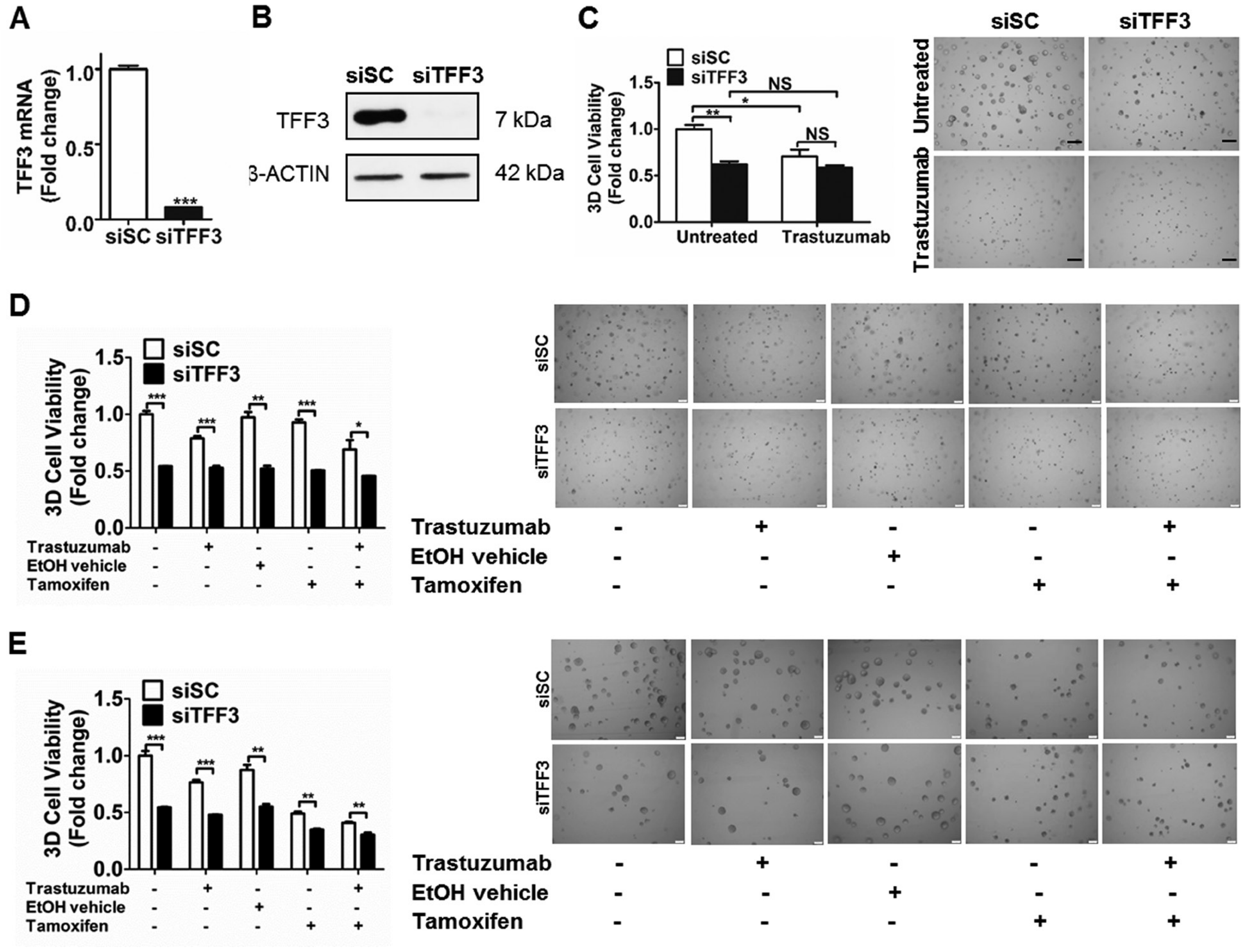

Supplementary Figure 4: Depletion of TFF3 decreased 3D Matrigel growth of HER2+/ER+ breast cancer cells treated with trastuzumab or the combination of trastuzumab and tamoxifen. BT474 cells were transiently transfected with siRNA against TFF3 or scrambled siRNA as control. TFF3 (**A**) mRNA and (**B**) protein levels in BT474-siSC and -siTFF3 cells were validated by qPCR and western blot with β-ACTIN as input control. (**C**) BT474-siSC and -siTFF3 cells were cultured in 5% FBS media containing 4% Matrigel and treated with 10 μg/ml trastuzumab over a period of 8 to 10 days. Cell viability in 3D Matrigel was measured by AlamarBlue assay. (**D**) BT474-siSC and -siTFF3 cells were cultured in 5% FBS media containing 4% Matrigel, and treated with 10 μg/ml trastuzumab, 5 μM tamoxifen or the combination over a period of 8 to 10 days. (**E**) MDA-MB-361-siSC and -siTFF3 cells were cultured in 10% FBS media containing 4% Matrigel, and treated with 10 μg/ml trastuzumab, 1 μM tamoxifen or the combination over a period of 8 to 10 days. Cell viability in 3D Matrigel was measured by AlamarBlue assay. Scale bar, 200 μm. *$p < 0.05$; **$p < 0.01$; ***$p < 0.001$.

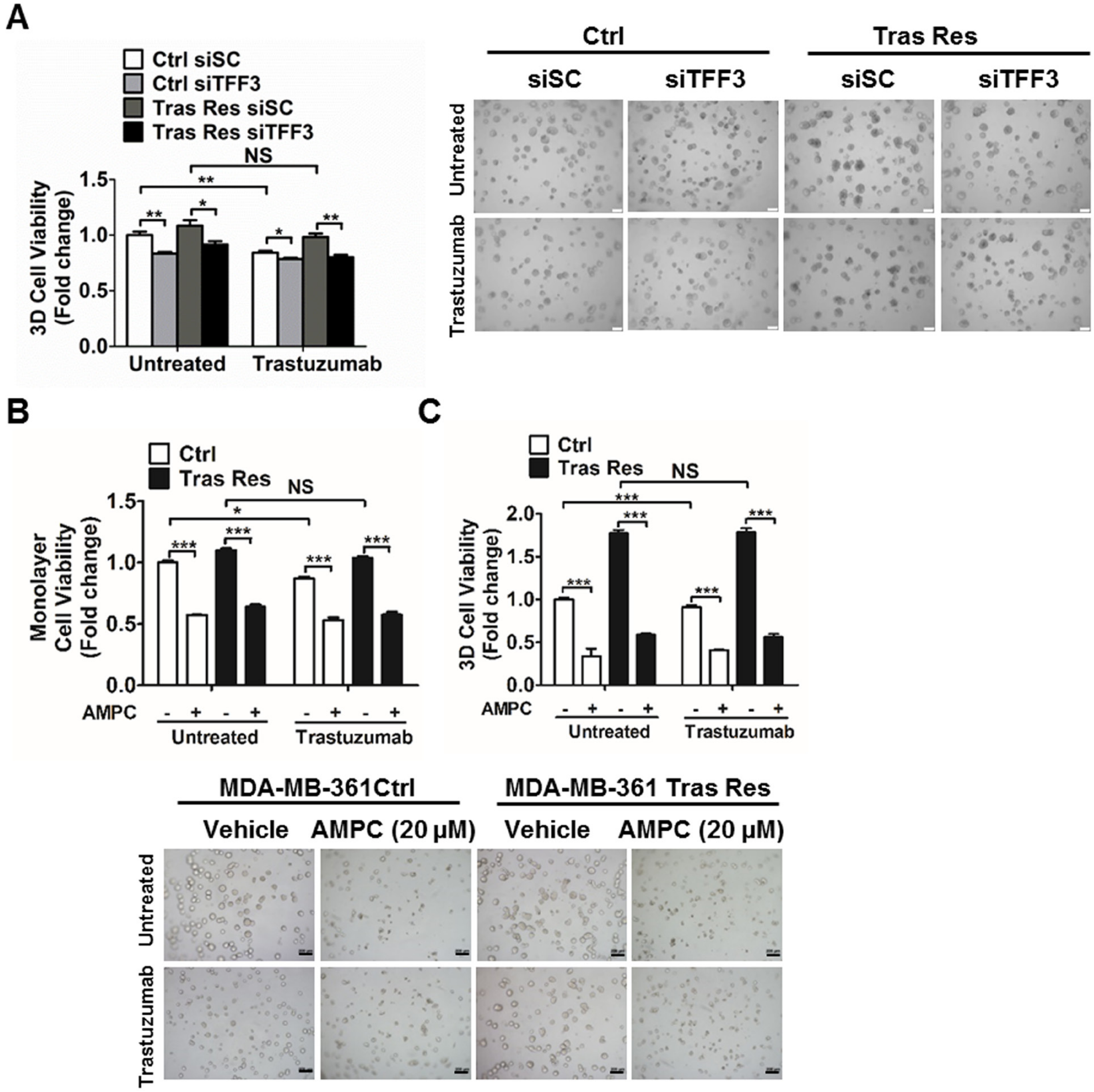

Supplementary Figure 5: Depletion or AMPC inhibition of TFF3 decreased the monolayer cell viability and 3D Matrigel growth of trastuzumab resistant MDA-MB-361 cells, without additional inhibitory effect from trastuzumab. (**A**) Control and trastuzumab resistant MDA-MB-361 cells transiently transfected with either scrambled or TFF3 siRNA were cultured in 10% FBS media containing 4% matrigel, and treated with 10 μg/ml trastuzumab over a period of 8 to 10 days. Cell viability in 3D Matrigel was measured by AlamarBlue assay. (**B**) Control and trastuzumab resistant MDA-MB-361 cells were treated with 20 μM AMPC ± 10 μg/ml trastuzumab in media supplemented with 10% FBS. Cell viability was measured by AlamarBlue assay after 6 days. (**C**) Control and trastuzumab resistant MDA-MB-361 cells were cultured in 10% FBS media containing 4% matrigel, and treated with 20 μM AMPC ± 10 μg/ml trastuzumab, over a period of 8 to 10 days. Cell viability in 3D Matrigel was measured by AlamarBlue assay. C or Ctrl: control cells; TR or Tras Res: trastuzumab resistant cells; Vehicle: DMSO solvent for AMPC. Scale bar, 200 μm. *$p < 0.05$; **$p < 0.01$; ***$p < 0.001$; NS, no significance.

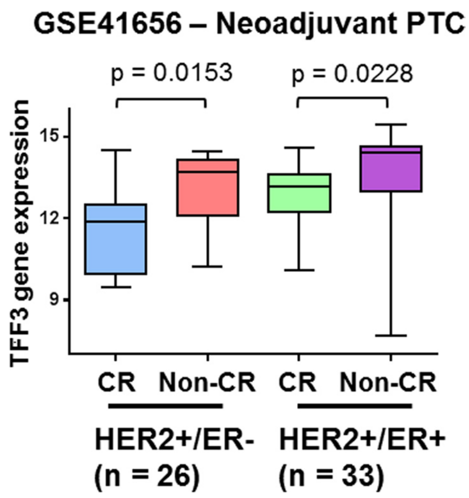

Supplementary Figure 6: Increased TFF3 expression is correlated with decreased response to a combination of trastuzumab and chemotherapy in HER2+ breast cancer patients. In the GSE41656 Neoadjuvant PTC (paclitaxel, trastuzumab, carboplatin) trial, TFF3 expression levels in complete responders (CR) and non-complete responders (non-CR) are compared among patients stratified according to their HER2 and ERα status.
